# Granular cell tumor of the brachial nerve in a dog: A case report

**DOI:** 10.29374/2527-2179.bjvm001424

**Published:** 2024-05-28

**Authors:** Kenichi Maeda, Seiichi Wada, Chiaki Shimaoka, Satomi Iwai, Shozo Okano

**Affiliations:** 1 Veterinarian, Kitasato University School of Veterinary Medicine, Department of Small Animal Surgery 2, Japan.; 2 Veterinarian, Kitasato University School of Veterinary Medicine, Veterinary Radiology, Japan.; 3 Veterinarian, Kitasato University Veterinary Teaching Hospital, Japan.

**Keywords:** canine, granular cell tumor, brachial nerve, canino, tumor de células granulares, nervo braquial

## Abstract

Here, we describe the first case of a granular cell tumor (GCT) derived from the brachial nerve. Eleven-year-old neutered female Chihuahua presented to the hospital with a bulge from the left neck to the axilla. The dog had a spherical subcutaneous mass on the cervical subcutis, and cytology hinted at adenocarcinoma or neuroendocrine tumor. However, the origin of the tumor remains unknown. During resection of the mass, bleeding was difficult to control owing to the high blood flow, and tumor removal was extremely difficult. The caudal aspect of the mass was attached to the brachial nerve and had to be removed, along with parts of the nerve fibers. The patient's postoperative course was fair, but it developed paralysis of the left thoracic limb. Pathology revealed that the mass was positive for S100 and vimentin, and GCT was diagnosed. Non-oral GCTs are extremely rare. The clinical diagnosis of GCT is difficult and is often confirmed histopathologically by excision. Although most cases of GCT are benign, they must be recognized as hemorrhagic, indistinct masses that mimic malignancy. Excision carries the risk of hemorrhage and damage to the surrounding tissues to secure margins.

## Resumo

Descrevemos aqui o primeiro caso de um tumor de células granulares (TCG) derivado do nervo braquial. Uma chihuahua castrada de 11 anos de idade deu entrada no hospital com uma protuberância do pescoço esquerdo até a axila. A cadela apresentava uma massa subcutânea esférica no subcutâneo cervical, e a citologia indicava adenocarcinoma ou tumor neuroendócrino. Entretanto, a origem do tumor permanece desconhecida. Durante a ressecção da massa, foi difícil controlar o sangramento devido ao alto fluxo sanguíneo, e a remoção do tumor foi difícil. O aspecto caudal da massa estava ligado ao nervo braquial e teve de ser removido, juntamente com partes das fibras nervosas. A evolução pós-operatória da paciente foi regular, mas ele desenvolveu paralisia do membro torácico esquerdo. O exame anatomopatológico revelou que a massa era positiva para S100 e vimentina, e o TCG foi diagnosticado. Os TCGs não orais são extremamente raros. O diagnóstico clínico do TCG é difícil e geralmente é confirmado histopatologicamente por excisão. Embora a maioria dos casos de TCG seja benigna, eles devem ser reconhecidos como massas hemorrágicas e indistintas que simulam malignidade. A excisão acarreta o risco de hemorragia e danos aos tecidos circundantes para garantir as margens.

## Introduction

Granular cell tumors (GCTs) were first described in humans in 1926 as granular myoblastomas (Abrikossoff, 1926). GCTs are closely related to schwannomas and are typically benign tumors derived from Schwann cells (Stefansson & Wollmann, 1982). In dogs, GCTs frequently occur in the oral cavity, particularly on the tongue (Patnaik, 1993) however, a few cases occurring in the central nervous system, such as intracranial (Higgins et al., 2001) or spinal cord cases (Rao et al., 2010), have been reported. Nonetheless, GCT occurring in peripheral nerves in humans is rarely reported (Vigier et al., 2014), and to the best of our knowledge, no case of primary GCT occurring in peripheral nerves has been reported in dogs. When treating tumors, not only GCT but also the tumor is identified using histology, and a surgical plan, including the extent of resection, should be devised. However, owing to the lack of clinical findings specific to GCT, the accuracy rate before surgery is extremely low (Battistella et al., 2014; Ferreira et al., 2017; van de Loo et al., 2015) and therefore, we must rely on pathological diagnosis after resection. Hence, when faced with an atypical GCT, as in this case, the surgeon must approach the case considering the possibility of various tumors without obtaining a definitive preoperative diagnosis. The purpose of this case report is to describe the first case of atypical GCT arising from the brachial plexus in a dog.

## Case report

An 11-year-old neutered female Chihuahua weighing 2.7 kg was presented to a nearby veterinary hospital 1 month after the owner noticed a subcutaneous mass on the neck and a slight decrease in appetite. Ultrasonography revealed a mass with abundant blood flow from the neck to the axilla, and malignancy was suspected. At the time of the initial evaluation, blood tests showed no abnormalities (Table 1), and radiographs showed a solitary tumor extending from the subcutaneous neck to the axilla, with no evidence of invasion into the surrounding area or notable metastasis to the lung field. Cytology suggested an epithelial tumor, such as an adenocarcinoma, and a CT scan and tissue biopsy were performed under general anesthesia for further evaluation.

**Table 1:** Results of the blood examination.

Test	Result	Reference value
Total Protein (g/dL)	7.7	5.6 ~ 7.5
Albumin (g/dL)	2.8	2.9 ~ 3.8
Glucose (mg/dL)	116	88 ~ 128
Cholesterol (mg/dL)	208	113 ~ 281
Aspartate Aminotransferase (IU/L)	19	14 ~ 42
Alanine Aminotransferase (IU/L)	23	15 ~ 79
Alkaline Phosphatase (IU /L)	269	50 ~ 316
Gamma-glutamyltransferase (IU /L)	7.8	4.0 ~ 12.0
Bilirubin (mg/dL)	0.00	0.00 ~ 0.13
Lactate Dehydrogenase (IU/L)	50	27 ~ 15
Creatinine (IU/L)	57	60 ~ 209
Phosphorus (mg/dL)	3.5	1.8 ~ 5.1
Calcium (mg/dL)	9.4	9.2 ~ 11.2
Urea nitrogen (mg/dL)	20.0	7.4〜37.0
Sodium (mEq/L)	148.6	142.0 ~ 152.
Potassium (mEq/L)	4.51	3.40 ~ 4.80
Chloride (mEq/L)	113	106.0 ~ 116.0
Red Blood Cells (10^6^/μL)	7.32	4.95~ 7.87
Hemoglobin (g/dL)	14.7	11.9〜18.9
Hematocrit (%)	42.6	35~ 57
White Blood Cells (10^3^/μL)	8.06	5.0 ~ 14.1

**Figure 1:**
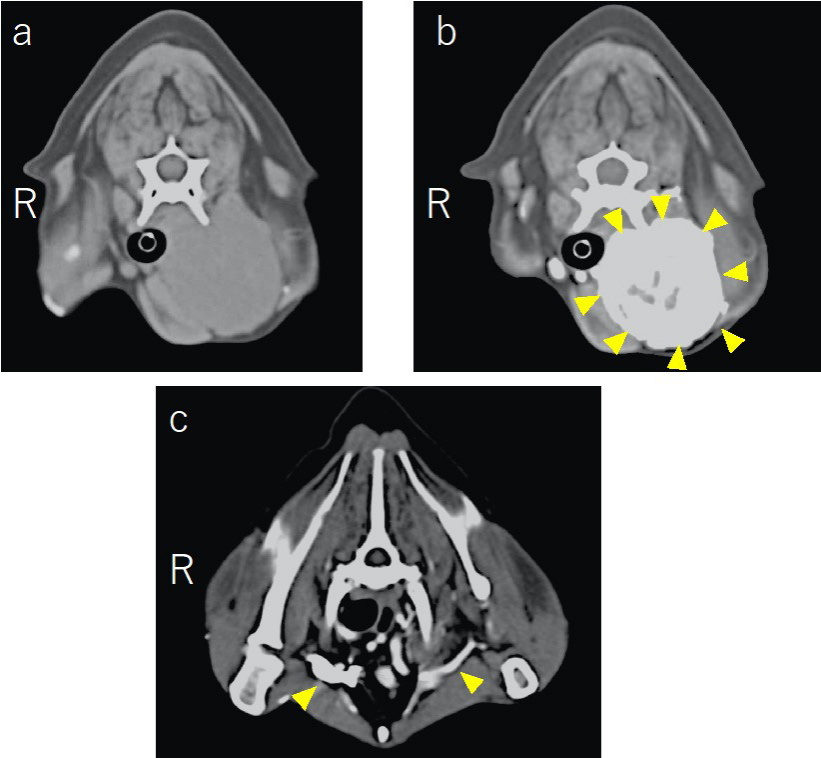
The results of CT scan. a: Before contrast agent injection a and b represent the transversal view of the C6 level. b: The tumor shows strong enhancement in the arterial phase of the CT scan (yellow arrowhead), indicating a massive distribution of arterial vessels in the tumor. c: Transverse view at the T2 level. Owing to compression by the tumor, the brachial vessels on the tumor side shrunk compared to those on the right side (yellow arrowhead).

**Figure 2:**
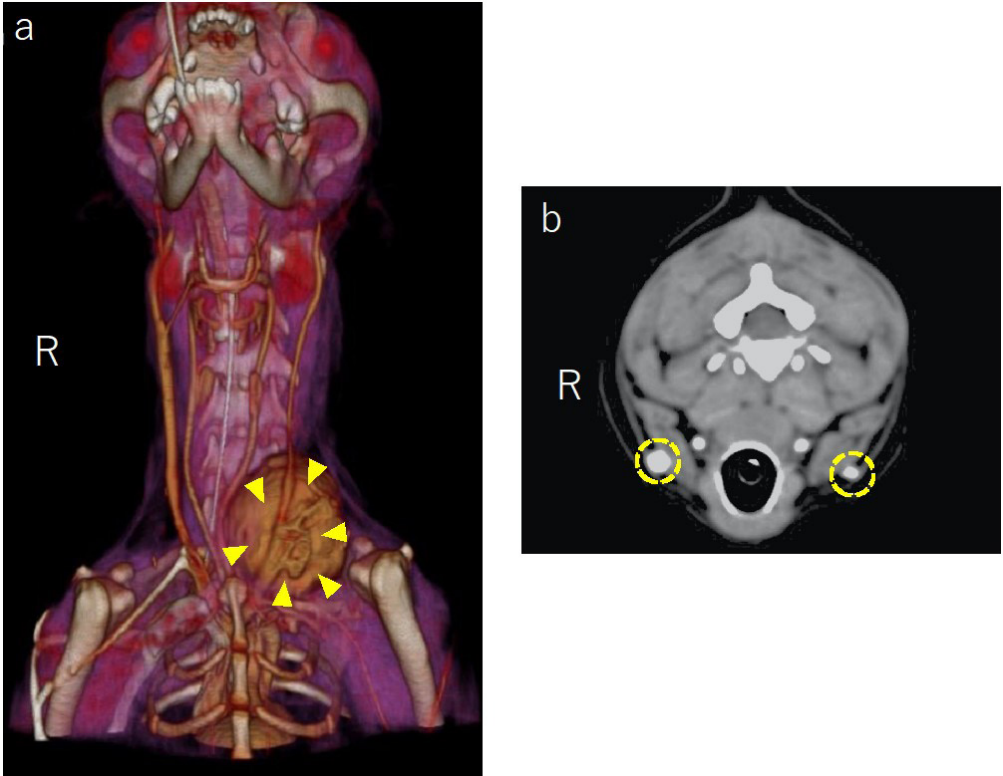
a: The reconstructed view 3D image of the head and neck region. The left cervical vein was attached to the mass (yellow arrowhead). b: Transverse view of the C5 level in the venous phase of the CT scan. The diameter of the right cervical vein was larger than that of the left (yellow dash circle).

CT revealed that the mass had developed subcutaneously in the neck, compressing the esophagus and the anterior thoracic chest wall. Blood flow in the mass was high and obstructed the blood flow in the axillary vein (Figure 1). In addition, the left jugular vein was trapped by the mass, and the blood flow was obstructed. As a result, blood flow to the contralateral (right) side would be excessive, and the diameter of the vessel would appear to be compensatory enlarged compared with that of the left (tumor) side (Figure 2).

Histological diagnosis was not confirmed by gross evaluation using hematoxylin and eosin staining. The cells showed a sheet-like proliferation of non-epithelial cells that were predominantly round, with slightly irregular nuclei and some unevenly sized vacuoles, presumably fat droplets, in the acidophilic, fine-granular cytoplasm. Cell division was barely visible at high magnification, and multiple capillaries were interspersed among the cells with no notable cellular atypia, making the histology atypical and difficult to identify. Granulomas, salivary gland, neuroendocrine, and granular cell tumors are all differential diagnoses. Additional immunostaining was positive for vimentin and MH and negative for AE1/AE3, PGP9.5, desmin, and CD204. Based on these results, the possibility of pheochromocytoma or well-differentiated liposarcoma was first considered, followed by a granuloma. Therefore, after consultation with the owner, it was decided to perform a wide excision despite the possibility of disability in the forelimbs, keeping the possibility of malignancy in consideration.

Anesthesia was induced via intravenous injection of fentanyl (0.005 mg/kg, Fentanyl Injection, Daiichi Sankyo Co., Ltd.), midazolam (0.1 mg/kg, Dorumicum Injection, Maruishi Pharmaceutical Co., Ltd.), and propofol (6 mg/kg, propofol 1% Injection, MSD Animal Health Co., Ltd.). General anesthesia was maintained with isoflurane (2–3%, Isoflurane, MSD Animal Health Co., Ltd.), and intraoperative analgesia was provided by continuous infusion of fentanyl (0.005–0.02 mg/kg/h).

With the patient in the supine position, an incision was made on the left side from the neck to the axilla. The subcutaneous tissue was bluntly dissected, and the mass was easily approached through the gap between the sternocleidomastoid and brachioradialis muscles. Adhesion between the mass and surrounding tissues was mild, but the jugular vein was trapped within the mass, which was consistent with the CT findings, and many blood vessels were distributed on the surface of the mass. The mass was detached from the surrounding tissues from the cephalic side using electrocautery and an ultrasonic coagulator; however, bleeding from the surface of the mass could not be adequately controlled, and the left jugular vein was ligated. However, the bleeding persisted, and achieving cephalad and dorsal dissection with repeated pressure hemostasis using hemostatic adrenaline was possible. However, the bleeding persisted, and there was hypotension due to the loss of circulating blood volume and compensatory tachycardia; therefore, the infusion volume was increased from 5 mL/kg/h to 10 mL/kg/h. The caudal side of the mass was adherent, and subsequent exploration confirmed and validated its attachment to the brachial plexus emerging from the fifth to eighth cervical vertebrae. Therefore, the patient underwent nerve resection to provide a safety margin despite the risk of forelimb paralysis.

After the bleeding stopped and no gross residual mass remained, the wound surface was infiltrated with 0.5% bupivacaine HCL (Marcaine; Sandoz Co., Ltd.) for analgesia, and the wound was closed. Although recovery from anesthesia was excellent and no clinical signs of blood loss, such as tachycardia or tachypnea, were observed, a whole blood transfusion of 20 mL/kg was initiated immediately after recovery from anesthesia. Postoperatively, fentanyl was administered for 8 h, after which buprenorphine (0.02 mg/kg, Lepetan injection, Otsuka Pharmaceutical Factory, Inc.) was administered intravenously every 8 h for 4 days for analgesia.

Postoperatively, the patient's appetite improved, and her general condition was good; however, knuckling of the left forelimb was observed.

**Figure 3:**
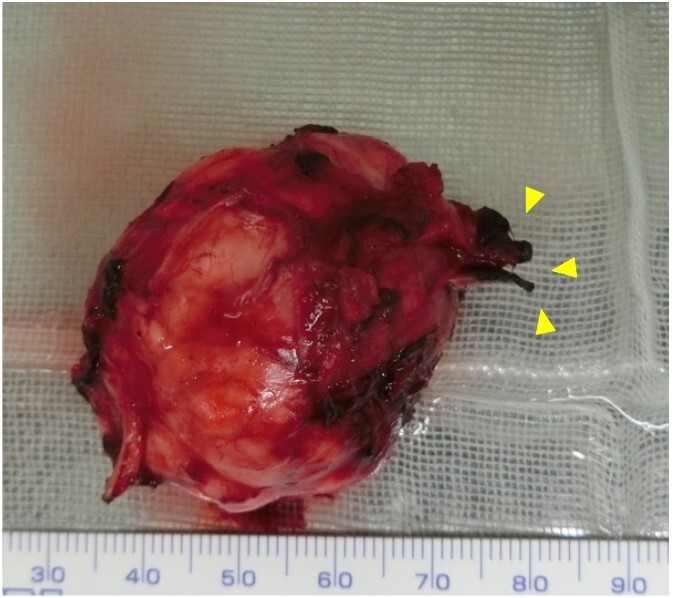
The excised mass was surrounded by a capsule, and nerve fibers (yellow arrowhead) were grossly visible.

**Figure 4:**
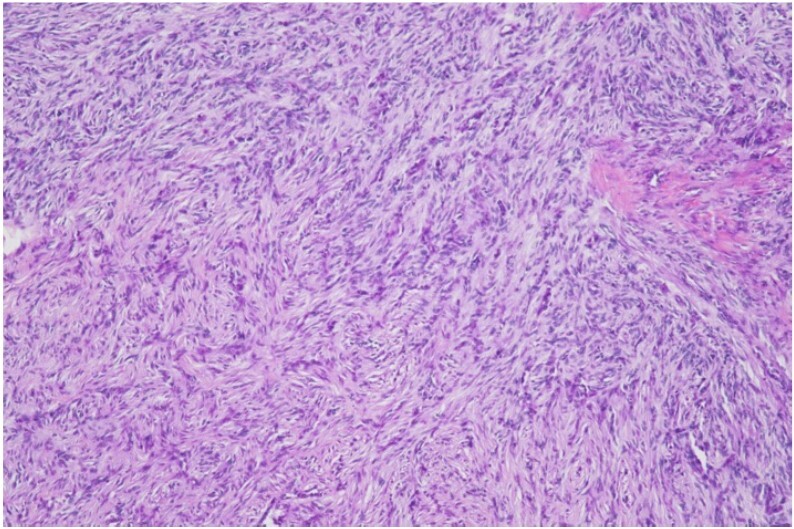
Hematoxylin Eosin stain pathologic view. There was sheet-like proliferation of non-epithelial cells that were round and partially spindle-shaped.

The excised mass was surrounded by a capsule, and nerve fibers were grossly visible (Figure 3). Pathological examination revealed that the excised neck tissue had a subcutaneous mass-like lesion with well-defined borders. Within the lesion was a sheet-like proliferation of non-epithelial cells that were round and partially spindle-shaped. These cells showed a mild nuclear size disparity, abundant acidophilic fine granular cytoplasm, and a peri-mass area containing nerve fibers. The tumor borders were relatively clear, and no tumor cells were found in the margins or within the vasculature (Figure 4).

**Figure 5:**
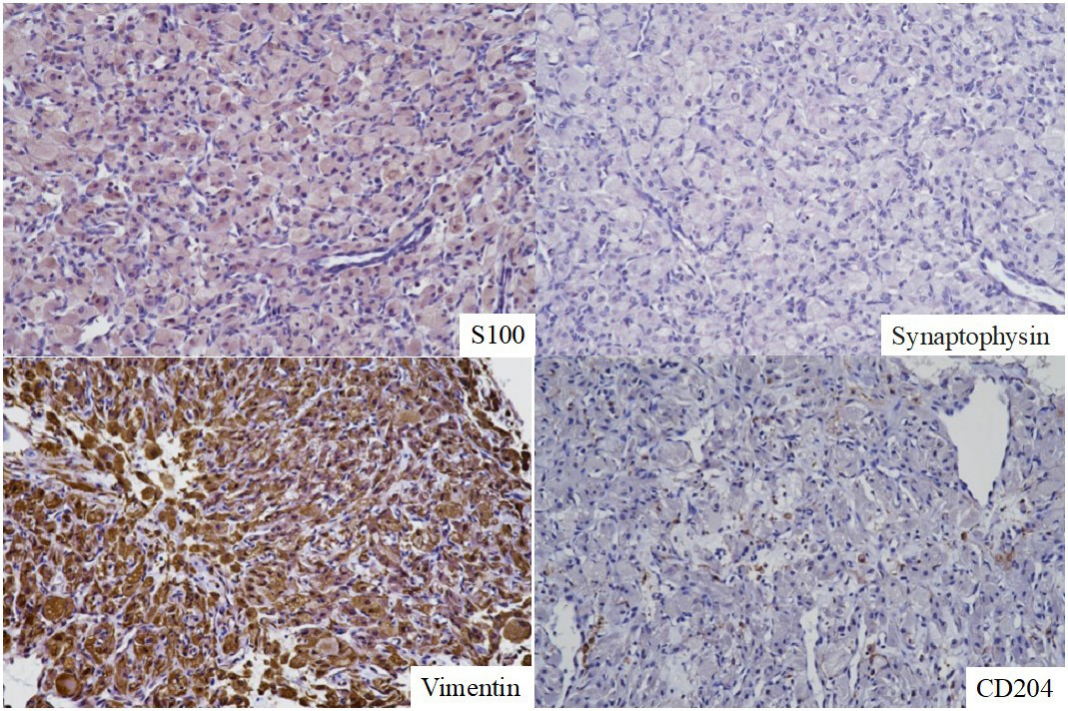
Results of the immune staining. Tumor cells were weakly positive for S100 and strongly positive for vimentin. No tumor cells were positive for synaptophysin or CD204.

**Figure 6:**
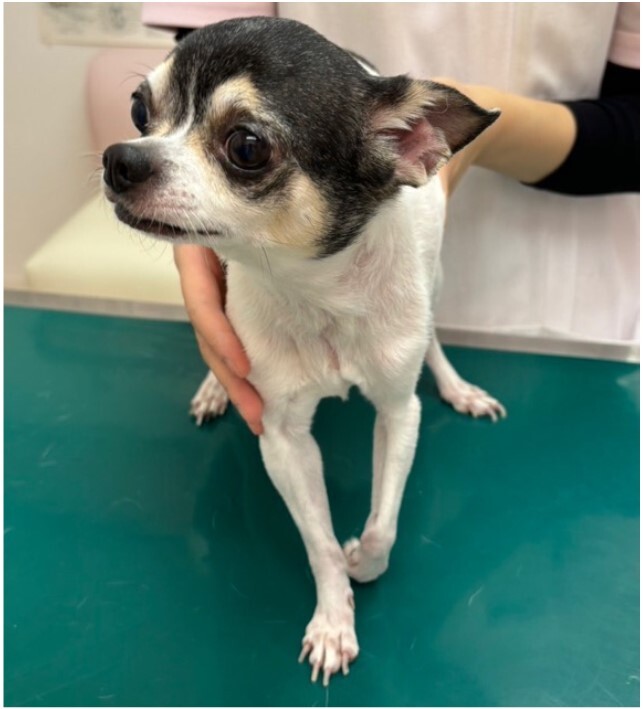
Six months after surgery. No recurrence was found but lameness of affected limb still existed. No Horner sign was observed.

The pathology of the resected tumor revealed that, despite the atypical site of origin and histology, the tumor was classified as a granular cell tumor based on the morphology of the tumor cells and the fact that immunostaining was positive for S100. The tumor was hypothesized to be of Schwann cell origin, and some parts of the tumor showed a Schwann cell tumor-like morphology (Figure 5). The tumor was resected with secure safety margins, and no tumor cells were found in the peripheral regions or the vasculature. Three months after surgery, paralysis of the forelimb persisted (Figure 6), and 6 months after surgery, paralysis persisted, and no symptoms of recurrence or metastasis were found.

## Discussion

The most common site for GCTs outside the oral cavity is the intracranial space (Higgins et al., 2001; Mayor et al., 2023). However, a literature search revealed that reports of GCTs in the peripheral nervous system are extremely rare in dogs, with only one case of euthanasia involving the left fourth lumbar spinal nerve (Rao et al., 2010). Therefore, in the present case, neither preoperative imaging, cytology, nor tissue biopsy could predict the presence of GCT. In addition to the extremely rare occurrence of GCT associated with peripheral nerves, as mentioned above, the difficulty of preoperative diagnosis of GCT. That is, GCTs lack specific clinical findings and cytomorphological features, and in this case, no obvious cellular atypia could be confirmed via preoperative tissue biopsy diagnosis, and it was difficult to identify the origin, followed by an immune histological diagnosis. The immunohistological diagnosis was positive for vimentin and MHC (II) and negative for AE1/AE3, PGP9.5, desmin, and CD204, and the possibility of pheochromocytoma or well-differentiated liposarcoma was considered first, followed by granuloma, but was not confirmed. Imaging revealed an abundant arterial blood flow within and around the mass. Immunohistological studies of vascular endothelial cells have been used to identify microvessels and calculate vascular density in various solid tumors and have shown a strong correlation between abnormal vascular density and patient prognosis (Srivastava et al., 1988; Toi et al., 1994). Therefore, although the origin of the mass in this case was unknown, it was determined to be malignant based on the abundant blood flow. When the tumor was resected, more than expected bleeding occurred, although it was predicted by imaging studies, and we had difficulty controlling it. In this case, the mass was large and tightly adhered to the surrounding tissue, making it difficult to identify the blood vessels entering the mass. However, bleeding was not controlled by dissecting the head of the mass and persisted until the brachial region was removed, suggesting that the main source of blood flow in the mass was the axillary artery. A branch of the brachial nerve was resected from C5 to C8 to remove the mass. GCTs involving the brachial nerve have not been reported in dogs, and only a few studies have been conducted in humans (Jia et al., 2017; Kim et al., 2012; Mindea et al., 2007). Kim et al. (2012) reported that the mass had invaded the brachial nerve, resulting in incomplete resection and recurrence. In principle, in the case of GCT, attempts are made to safely remove the mass microsurgically by skeletonizing it from the infiltrated nerve; however, depending on the degree of infiltration, this may be difficult and inevitably results in some degree of nerve damage that affects upper extremity function. This is of great concern, especially in humans, because of its impact on the patient’s postoperative social activities. In the present case, it was predicted that the mass would be associated with the brachial nerve before surgery, and the owner was informed and consented to the possibility of forelimb paralysis due to resection of the mass. Furthermore, as the goal was to achieve a wide and complete resection of the mass, considering that it was a malignant tumor, complete resection was achieved by resecting the mass, including the nerves. Postoperative neurological examination revealed loss of proprioceptive sensation in the forelimb, suggesting damage to nerve fibers in the C5 to C8 region. However, no Horner sign was observed, suggesting no deep dysfunction, such as nerve root entrapment, had occurred.

Similar to other tumor types, the treatment of localized GCTs, both benign and malignant, begins with surgical excision. Although there are no reports of prognosis in dogs, such as recurrence after peripheral nerve GCT surgery, authors expelienced the benign GCT occurring on the surface of the tongue rapidly recurred several weeks after surgery. In humans, in cases of benign GCTs in which wide local excision is performed, there is 2–8% recurrence with negative margins and >20% recurrence with positive margins. GCTs are generally benign lesions that can be safely managed with local excision on clear margins (Finkel & Lane, 1982; Khansur et al., 1987). Radiation and chemotherapy are not recommended because of the high degree of tumor resistance (Lack et al., 1980). Hence, the initial surgery is crucial for securing safety margins.

GCTs are rare in the brachial plexus region and can manifest in many ways depending on the site of involvement. GCTs of the brachial plexus may be misdiagnosed before histological confirmation because they present with subtle clinical symptoms, and CT findings are often similar to those of other nerve sheath tumors.

## Conclusions

In the present case, we could not make a definitive diagnosis before surgery because the tumor mimicked other tumors. Surgical treatment is imperative because total resection of the tumor is the only way to reduce the frequency of recurrence and metastasis, as cell tumors are refractory to conventional treatment. This report provides veterinarians with a diagnosis and treatment options for tumors of unknown origin before surgery.
